# Comparing the Outcomes of Cefoperazone/Sulbactam-Based and Non-Cefoperazone/Sulbactam-Based Therapeutic Regimens in Patients with Multiresistant *Acinetobacter baumannii* Infections—A Meta-Analysis

**DOI:** 10.3390/antibiotics13090907

**Published:** 2024-09-23

**Authors:** Chienhsiu Huang, Lichen Lin, Sufang Kuo

**Affiliations:** 1 Department of Internal Medicine, Dalin Tzu Chi Hospital, Buddhist Tzu Chi Medical Foundation, Chiayi 62247, Taiwan; 2 Department of Nursing, Dalin Tzu Chi Hospital, Buddhist Tzu Chi Medical Foundation, Chiayi 62247, Taiwan; df462594@tzuchi.com.tw (L.L.); dl08596@tzuchi.com.tw (S.K.)

**Keywords:** *Acinetobacter baumannii*, cefoperazone, sulbactam, mortality, multiresistant

## Abstract

The addition of sulbactam restores the complete range of cefoperazone activity against bacteria and extends its spectrum of action to include the *Acinetobacter* species. The effectiveness of cefoperazone/sulbactam against multiresistant *Acinetobacter baumannii* has not been investigated. The purpose of the current meta-analysis was to compare the efficacy of cefoperazone/sulbactam-based therapeutic regimens and non-cefoperazone/sulbactam-based therapeutic regimens in the treatment of multiresistant *Acinetobacter baumannii* infections. The current meta-analysis of 10 retrospective studies provides evidence that cefoperazone/sulbactam-based therapeutic regimens are superior to non-cefoperazone/sulbactam-based therapeutic regimens in terms of 30-day mortality and clinical improvement in patients with multiresistant *Acinetobacter baumannii* infections. The risk of mortality was reduced by 38% among multiresistant *Acinetobacter baumannii* infections in patients who received cefoperazone/sulbactam-based therapeutic regimens. The cefoperazone/sulbactam-based combination therapy was superior to the cefoperazone/sulbactam monotherapy in terms of 30-day mortality when both therapeutic regimens were compared to the tigecycline monotherapy in patients with multiresistant *Acinetobacter baumannii* infections.

## 1. Introduction

*Acinetobacter baumannii* is a common hospital-acquired pathogen found worldwide that can cause serious infections such as urinary tract infections, bacteremia, meningitis, pneumonia, and infections linked to catheters [1,2]. For *Acinetobacter baumannii* infections, carbapenem antibiotics are the usual course of therapy due to carbapenem’s antibacterial action and the reduced frequency of adverse effects. However, there are now significant obstacles facing the treatment approaches due to a global rise in carbapenem-resistant *Acinetobacter baumannii* (CRAB). According to the surveillance data, up to 85% of *Acinetobacter baumannii* strains in Latin America and 88% of *Acinetobacter baumannii* strains in Europe have high resistance to carbapenems [3,4]. According to a nationwide survey conducted in China, the prevalence of CRAB rose from 31.0% in 2005 to 66.7% in 2014 [5]. For CRAB infections, there is no established “standard of care” antibiotic regimen against which to compare the efficacy of different treatment plans. There are few reliable studies comparing the efficacy of routinely used antimicrobial agents; thus, there is an insufficient amount of data to support the additive benefit of routinely used combinations for CRAB infections. The World Health Organization identified CRAB as one of the infections that most required novel medications in 2017.

Cefoperazone is a broad-spectrum cephalosporin and exhibits good efficacy against *Pseudomonas aeruginosa*, *Escherichia coli*, and species of *Klebsiella*, *Enterobacter*, and *Proteus* when β-lactamases are absent from the bacteria.

The study by Chiang TT et al. showed that a combination of cefoperazone and sulbactam is effective against important Gram-negative bacteria, including extended-spectrum β-lactamase-producing *Escherichia coli* and *Klebsiella pneumoniae*. In addition, the combination also exhibited better activity against *Acinetobacter baumannii* than cefoperazone alone (71.2% versus 0.27%) [6]. The study by Chang PC et al. showed that cefoperazone/sulbactam at a 1:1 ratio had a susceptibility rate against carbapenem-resistant *Escherichia coli* (67.6%) and carbapenem-resistant *Acinetobacter baumannii* (68.0%) [7]. According to Choi et al., patients with *Acinetobacter baumannii* bacteremia who received cefoperazone/sulbactam had a lower 30-day mortality rate [7/35 (20%)] than those who received imipenem/cilastatin [vs. 6/12 (50%), *p* = 0.065] [8]. Xia et al. found that among patients with hospital-acquired pneumonia due to CRAB, those who received cefoperazone/sulbactam alone or in combination with antimicrobial medications had a 30-day survival rate of 95.1%, whereas those who did not (*p* < 0.05) had a rate of 73.3% [9]. The effectiveness of cefoperazone/sulbactam against multiresistant *Acinetobacter baumannii* has not been investigated in a clinical context. The purpose of this current meta-analysis was to compare the efficacy of cefoperazone/sulbactam-based therapeutic regimens and non-cefoperazone/sulbactam-based therapeutic regimens in an effort to overcome this limitation and better understand the effectiveness of cefoperazone/sulbactam-based regimens in the treatment of multiresistant *Acinetobacter baumannii* infections.

## 2. Relevant Sections

### 2.1. Data Search Strategy

The literature search was performed using the PubMed, Web of Science, and Cochrane Library databases to identify all clinical studies and meta-analyses or systematic reviews on the topic from 1 January 2000 to 31 October 2023. In the databases, we used the following search string: (cefoperazone OR sulbactam OR cefoperazone/sulbactam) (carbapenem-resistant *Acinetobacter baumannii* infection or CRAB infection or multidrug-resistant *Acinetobacter baumannii* infection or MDRAB infection or extensively drug-resistant *Acinetobacter baumannii* infection or XDRAB infection). Previously published systematic reviews and meta-analyses were reviewed to identify any additional studies that may have been missed in the primary literature search. Only articles in English were included.

### 2.2. Selection Criteria

Any randomized controlled trial, controlled clinical study, or cohort study that compared the clinical efficacy of cefoperazone/sulbactam-based therapeutic regimens (including cefoperazone/sulbactam monotherapies and combination therapies with cefoperazone/sulbactam-based therapeutic regimens) against non-cefoperazone/sulbactam-based therapeutic regimens for the treatment of a multiresistant *Acinetobacter baumannii* infection was eligible to be included in the meta-analysis. The meta-analysis did not include in vitro research or studies that examined the antimicrobial susceptibility of clinical isolates or bacterial strains in the absence of clinical data.

### 2.3. Data Extraction

Two unbiased reviewers gathered data from the relevant research. Disagreements were settled by a third reviewer. The first author’s name, the publication year of the study, the type of study, the number of participants in each treatment group, the dosage of cefoperazone/sulbactam, the drug given with cefoperazone/sulbactam, the comparator drug, the clinical improvement rate, and the 30-day mortality or hospital mortality rate were all taken from studies that satisfied the inclusion criteria.

### 2.4. Definition

The primary outcome was hospital mortality (or 30-day mortality). Hospital mortality was death by all causes before patient discharge. Thirty-day mortality was mortality occurring within 30 days of an index event, such as admission or leaving the hospital. The secondary outcome was clinical improvement, which was defined as a complete or partial resolution (improvement) of the symptoms or signs related to *Acinetobacter baumannii* infection by the end of the therapy. Multidrug-resistant (MDR) refers to nonsusceptibility to ≥1 agent in ≥3 antimicrobial categories. Extensive drug resistance (XDR) refers to nonsusceptibility to ≥1 agent in all but ≤2 antimicrobial categories. Nonsusceptibility to all antimicrobial agents tested was defined as pandrug resistance (PDR) [10].

### 2.5. Quality Assessment

The risk of bias in nonrandomized studies of interventions (ROBINS-I) tool was used to evaluate the observational studies [11]. We conducted a sensitivity analysis by systematically removing each study and assessed the impact of the study quality on the effect estimates. The quality of the evidence was ranked based on the risk of bias according to the Grading of Recommendations Assessment, Development and Evaluation (GRADE) approach at the outcome level [12,13].

### 2.6. Statistical Analysis

RevMan 5 (https://training.cochrane.org/online-learning/core-software/revman) (accessed on 5 July 2024) and Cochrane Review Manager software were used to conduct the statistical analyses. Fixed effects and random effects were utilized for the data analysis. Statistical heterogeneity was evaluated using the Q-test and I^2^ statistical techniques. Significant heterogeneity between the studies was defined as an I^2^ greater than 50% and a *p*-value for the Q-test less than 0.10 for each study. We tabulated the study intervention features and compared them to the scheduled groups for each synthesis using forest plots. The funnel plot was examined to determine the degree of publication bias.

## 3. Results

**Figure 1:**
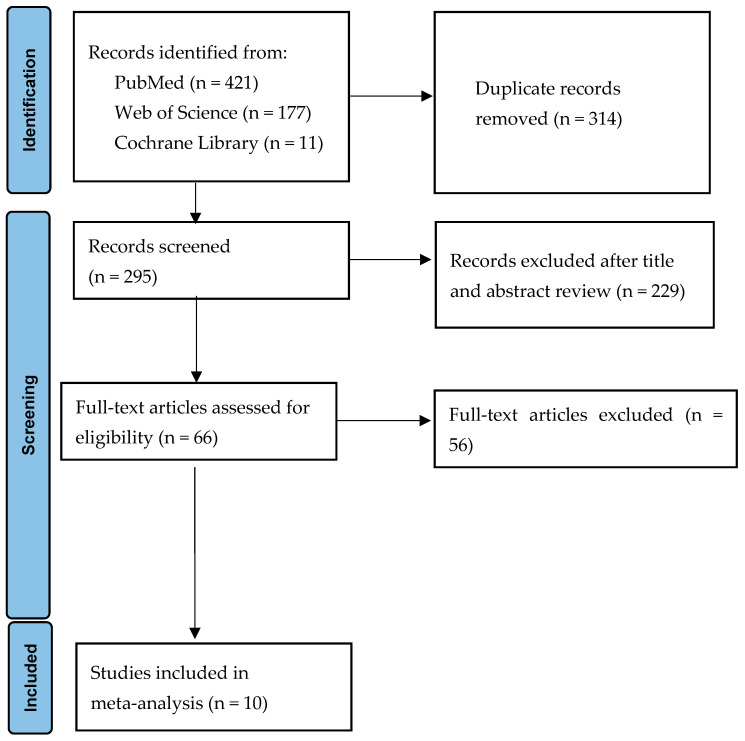
Flow diagram of the study selection process.

The details of the study selection process are shown in Figure 1. The numbers of studies from the initial search results from PubMed, Web of Science, and the Cochrane Library were 421, 177, and 11, respectively. There were 314 duplicate articles. A total of 229 irrelevant studies were identified by reading the title and/or abstract. After excluding the duplicates and irrelevant studies, 66 potentially relevant articles remained. After a full-text article review, 56 articles were excluded because they lacked results comparing the outcomes of cefoperazone/sulbactam-based and non-cefoperazone/sulbactam-based therapeutic regimens in adults with multiresistant *Acinetobacter baumannii* infections. Finally, 10 studies were included in the meta-analysis [9,14,15,16,17,18,19,20,21,22]. All studies were retrospective studies. The main characteristics of the 10 included studies are shown in Table 1. All of the studies had a high risk of bias (Table 2).

**Figure 2:**
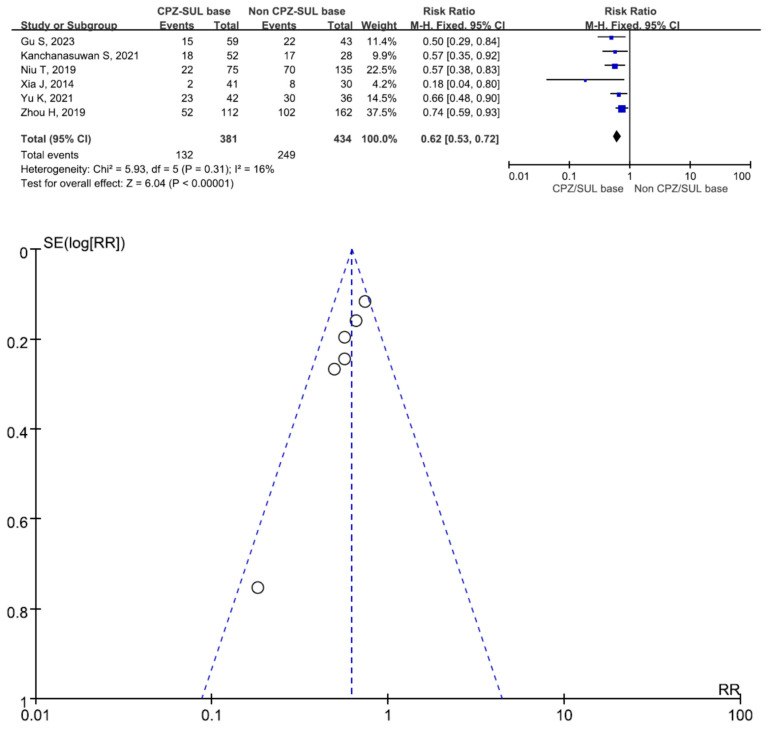
The 30-day mortality rate between patients treated with cefoperazone/sulbactam-based therapeutic regimens and those treated with non-cefoperazone/sulbactam-based therapeutic regimens. (Inverted funnel plots revealed a symmetric relationship) [9,17,18,20,21,22].

**Figure 3:**
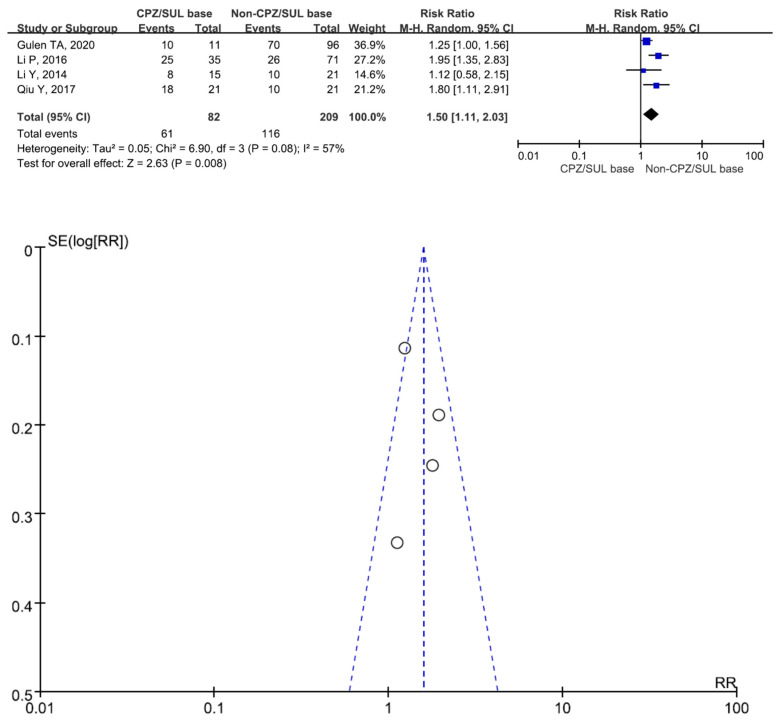
The clinical improvement between patients treated with cefoperazone/sulbactam-based therapeutic regimens and those treated with non-cefoperazone/sulbactam-based therapeutic regimens. (Inverted funnel plots revealed a symmetric relationship) [14,15,16,19].

**Figure 4:**
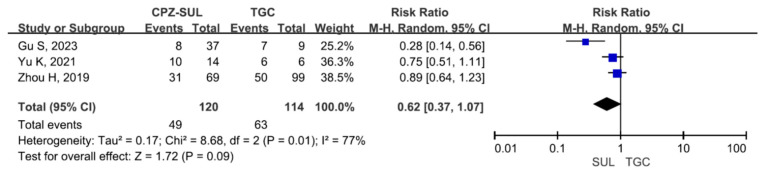

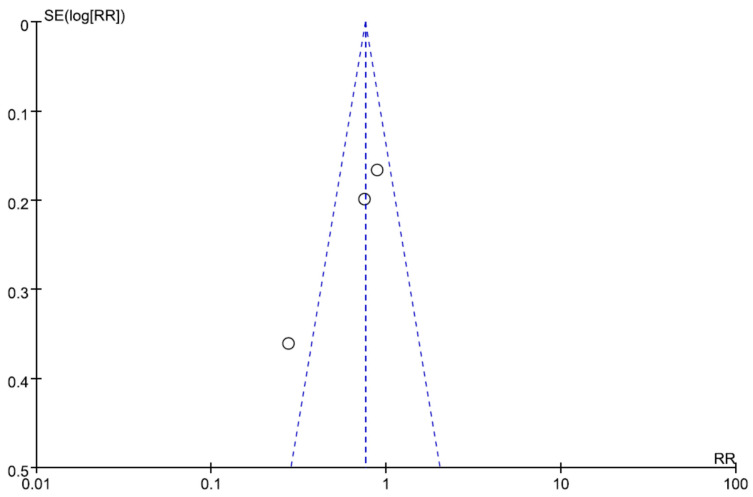
The 30-day mortality rate between patients treated with cefoperazone/sulbactam monotherapy and those treated with tigecycline monotherapy. (Inverted funnel plots revealed a symmetric relationship) [17,20,22].

**Figure 5:**
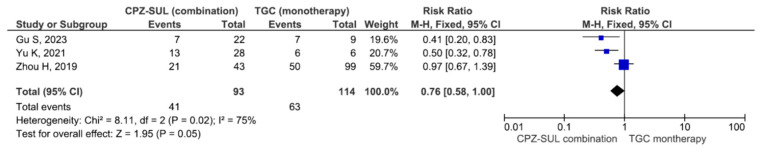

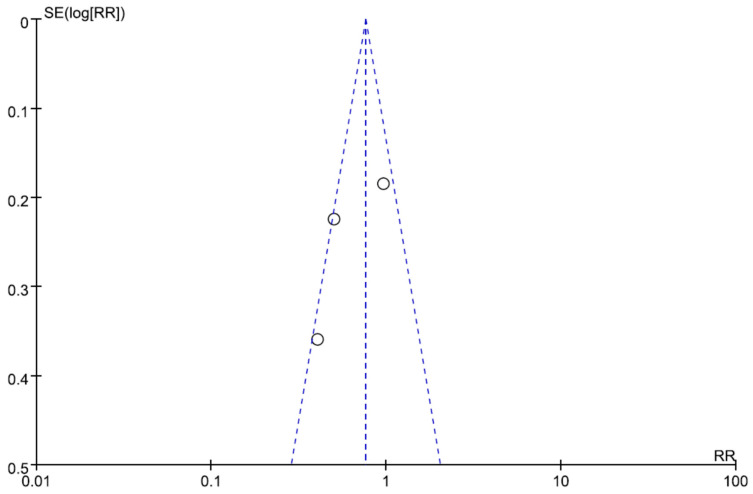
The 30-day mortality rate between patients treated with cefoperazone/sulbactam-based combination therapy and those treated with tigecycline monotherapy. (Inverted funnel plots revealed a symmetric relationship) [17,20,22].

There were 463 patients in the cefoperazone/sulbactam-based therapeutic regimen group and 643 patients in the non-cefoperazone/sulbactam-based therapeutic regimen group. Six studies involving 815 patients (381 receiving cefoperazone/sulbactam-based therapeutic regimens and 434 receiving non-cefoperazone/sulbactam-based therapeutic regimens) reported a 30-day mortality (or hospital mortality) [9,17,18,20,21,22]. There was a statistically significant difference in the 30-day mortality (or hospital mortality) between the patients treated with cefoperazone/sulbactam-based therapeutic regimens and those treated with non-cefoperazone/sulbactam-based therapeutic regimens (RR = 0.62, 95% CI = 0.53–0.72, *p* < 0.001, I^2^ = 16%) (Figure 2). Four studies involving 291 patients (82 receiving cefoperazone/sulbactam-based therapeutic regimens and 209 receiving non-cefoperazone/sulbactam-based therapeutic regimens) reported clinical improvement [14,15,16,19]. There was a statistically significant difference in the clinical improvement between patients treated with the cefoperazone/sulbactam-based therapeutic regimens and those treated with the non-cefoperazone/sulbactam-based therapeutic regimens (RR = 1.50, 95% CI = 1.11–2.03, *p* = 0.008, I^2^ = 57%) (Figure 3). Three studies involving 234 patients (120 receiving cefoperazone/sulbactam monotherapy and 114 receiving tigecycline monotherapy) reported a 30-day mortality (or hospital mortality) [17,20,22]. There was no statistically significant difference in the 30-day mortality (or hospital mortality) between the patients treated with the cefoperazone/sulbactam monotherapy and those treated with the tigecycline monotherapy (RR = 0.62, 95% CI = 0.37–1.07, *p* = 0.09, I^2^ = 77%) (Figure 4). Three studies involving 207 patients (93 receiving cefoperazone/sulbactam-based combination therapy and 114 receiving tigecycline monotherapy) reported a 30-day mortality (or hospital mortality) [17,20,22]. There was a statistically significant difference in the reported 30-day mortality (or hospital mortality) between the patients treated with cefoperazone/sulbactam-based combination therapy and those treated with tigecycline monotherapy (RR = 0.76, 95% CI = 0.58–1.00, *p* < 0.001, I^2^ = 0%) (Figure 5).

## 4. Discussion

To our knowledge, this is the first meta-analysis that has evaluated the efficacy of cefoperazone/sulbactam-based and non-cefoperazone/sulbactam-based therapeutic regimens in patients with multiresistant *Acinetobacter baumannii* infections. The current meta-analysis of 10 retrospective observational studies provides evidence that the cefoperazone/sulbactam-based therapeutic regimens are superior to the non-cefoperazone/sulbactam-based therapeutic regimens in terms of 30-day mortality and clinical improvement in patients with multiresistant *Acinetobacter baumannii* infections. The cefoperazone/sulbactam-based combination therapy is superior to the cefoperazone/sulbactam monotherapy in terms of 30-day mortality when both therapeutic regimens are compared to the tigecycline monotherapy in patients with multiresistant *Acinetobacter baumannii* infections. The current meta-analysis recommends cefoperazone/sulbactam-based therapeutic regimens in conjunction with one additional antimicrobial agent for the treatment of multiresistant *Acinetobacter baumannii* infections.

Although colistin has strong in vitro efficacy against strains of *Acinetobacter baumannii*, its restricted therapeutic range, absence of clinically meaningful susceptibility breakpoints, and severe nephrotoxicity and neurotoxicity are drawbacks [23]. Colistin has demonstrated therapeutic effectiveness in the management of urinary tract, wound, and bloodstream infections. Positive results have also been recorded using combination therapy with intravenous colistin and nebulized colistin for pneumonia, despite the intravenous form of the drug having very low lung penetration. Strains with colistin-heteroresistance have been shown to generate resistance during therapy. However, identifying these strains using standard susceptibility testing procedures is difficult, and the current rates of resistance may be underestimated [24,25,26]. There is also some uncertainty about the colistin dose. Markou et al. discovered that standard dosing regimens for critically ill adult patients were linked with suboptimal peak concentrations/minimum inhibitory concentrations [27]. Colistin-based treatments for multiresistant *Acinetobacter baumannii* infections are by no means secure or efficient. Since 2006, tigecycline—which was created to treat multidrug-resistant infections and has strong in vitro action against *A. baumannii*—has been used to treat CRAB infections [28]. Tigecycline is approved and effective in treating complicated skin and soft-tissue infections, intra-abdominal infections, and community-acquired bacterial pneumonia. Tigecycline is often active when in vitro against multidrug-resistant *Acinetobacter baumannii* (MDRAB); furthermore, it has been used to treat infections caused by *Acinetobacter baumannii* in numerous different locations, such as bloodstream and respiratory infections, because there are no effective alternatives. Because of the enhanced tissue penetration, which can result in serum concentrations much below the pharmacodynamic breakpoint and cause recurrent bacteriemia and, in certain situations, the fast evolution of resistance, the use of tigecycline for bloodstream infections is particularly contentious [29,30,31]. These discouraging findings are consistent with the findings of previous studies [32,33,34]. These factors, together with rising tigecycline resistance rates among CRAB, make the usage of tigecycline unfavorable. Therefore, the available antimicrobials (tigecycline and colistin) have numerous pharmacokinetic drawbacks, including a narrow therapeutic spectrum, low plasma concentration, emerging resistance, and severe toxicity, which limits the therapeutic options for CRAB infections [35,36,37,38].

Sulbactam is an irreversible, competitive β-lactamase inhibitor that saturates PBP1a/1b and PBP3 of the isolates of *Acinetobacter baumannii* in high dosages [39]. The in vitro investigations by Beganovic M et al. demonstrated that a significant kill rate against CRAB was achieved by using a triple treatment, which included high dosage minocycline (700 mg load, followed by 350 mg Q12h), a continuous infusion of sulbactam (9 g/24 h), and polymyxin B (2.5 mg/kg Q12h). There was no regrowth and little resistance development [40]. For *Acinetobacter*-derived cephalosporinases (Class C) and oxacillinases (Class D) enzymes generated by CRAB, sulbactam serves as a substrate. High-dose sulbactam increases the likelihood that sulbactam successfully reaches its PBP targets. Durlobactam effectively inhibits the class C and D enzymes, and enabling lower doses of sulbactam is more likely to successfully reach its PBP targets with the protection of durlobactam [41,42]. Jaruratanasirikul et al. evaluated the best sulbactam dosage schedules and showed that sulbactam could be a good alternative for treating critically ill patients with severe sepsis caused by MDRAB. They suggested that a 3 g/day dose of sulbactam would be an alternative treatment option for less susceptible pathogens, such as MDRAB [43,44]. According to the study by Liu J et al., when compared to other therapeutic regimens (including colistin and rifampicin, colistin and fosfomycin, colistin with sulbactam, colistin monotherapy, colistin with two additional antibacterial agents, colistin with carbapenems, and sulbactam with carbapenems) in patients with MDRAB or XDRAB infections, the therapeutic regimen of high-dose sulbactam combined with one additional antibacterial agent (levofloxacin, minocycline, or tigecycline) led to the most clinical improvement, but there were no statistically significant differences in all-cause mortality. Liu J et al. concluded that the combination regimens with high-dose sulbactam (≥4 g/day) appear to be more successful than those containing colistin in treating MDRAB and XDRAB infections, so combination regimens with high-dose sulbactam could be the best option overall [45]. According to the study by Deng Y et al., for patients with MDRAB pneumonia, patients treated with regimens containing tigecycline, carbapenem, and sulbactam had a much lower mortality rate (18.1%) than those treated with regimens containing tigecycline plus carbapenem (54.5%) (*p* < 0.001). Combination treatment with tigecycline and sulbactam may help lower the mortality rate among patients suffering from severe hospital-acquired pneumonia caused by MDRAB. Furthermore, high-dose sulbactam (>3 g/day) might be preferable to low-dose sulbactam (≤3 g/day) [46]. Ampicillin/sulbactam is a reasonably priced substitute for imipenem and is at least as effective in treating nonlife-threatening CRAB infections [47]. Clinical outcome data support the use of high-dose ampicillin/sulbactam (total daily dose of 6–9 g of the sulbactam component) as a component of combination therapy for multiresistant *Acinetobacter baumannii* infections [48,49,50,51]. For the treatment of CRAB infections, the Infectious Diseases Society of America recommends utilizing high-dose ampicillin/sulbactam (a total daily dosage of 6–9 g of the sulbactam component) in conjunction with at least one additional drug. Polymyxin B, minocycline, tigecycline, or cefiderocol are other drugs that may be used in combination regimens for the treatment of CRAB infections. The use of rifampin, imipenem/cilastatin, meropenem, or fosfomycin is not recommended as part of a combination treatment [52].

Currently, numerous β-lactam/β-lactamase inhibitor antibiotics, such as amoxicillin/clavulanate, ampicillin/sulbactam, piperacillin/tazobactam, cefoperazone/sulbactam, ceftolozane/tazobactam, ceftazidime/avibactam, and meropenem/vaborbactam, have demonstrated synergistic in vitro activity against multidrug-resistant organisms [53,54]. Wang L et al. showed that a stepwise increase in the ratio of sulbactam to partner β-lactam antibiotics led to a stepwise decrease in the MICs and a stepwise increase in susceptibility. The susceptibility rates for imipenem/sulbactam 1:3, ampicillin/sulbactam 1:3, and cefoperazone/sulbactam 1:3 reached 16.3%, 58.3%, and 91%, respectively. The increasing proportion of sulbactam could enhance the antimicrobial activities of imipenem/sulbactam, ampicillin/sulbactam, and cefoperazone/sulbactam combinations against *A. baumannii*, with cefoperazone/sulbactam being the most potent combination. Cefoperazone/sulbactam 1:3 displayed the best in vitro activity among all sulbactam-based combinations. The authors suggested that a high-ratio cefoperazone/sulbactam treatment could be a promising antimicrobial regimen for *Acinetobacter baumannii* infection [55].

A higher dosage of sulbactam does not always result in more antibacterial activity. Nevertheless, what ratio of cefoperazone to sulbactam inhibits multiresistant *Acinetobacter baumannii* the most remains unknown. There are several in vitro activity studies that investigate cefoperazone/sulbactam against multiresistant *Acinetobacter baumannii* in the literature. According to Ku YH et al., in vitro tests show that cefoperazone/sulbactam (1:1 or 1:2) is more effective against most multiresistant organisms, including ESBL- and AmpC-producing Enterobacteriaceae and CRAB. The available data support the use of a 2:1 ratio of cefoperazone/sulbactam (e.g., 2 g:1 g) as the ideal way to treat sulbactam-susceptible CRAB, as large doses of the inhibitor are not needed with such a low sulbactam MIC. Patients infected with the non-carbapenemase-mediated CRAB should be treated with cefoperazone/sulbactam in a 1:1 or 1:2 ratio (e.g., 2 g:2 g or 1 g:2 g). On the other hand, increased sulbactam concentrations may induce AmpC production. Carbapenem-resistant *Pseudomonas aeruginosa* was not affected by increasing the sulbactam composites of cefoperazone/sulbactam. This suggests that the presence of carbapenemases or AmpC overproduction could not be overcome by increasing the sulbactam levels to recover cefoperazone activity [56]. The in vitro activity study by Chang PC et al. showed that the novel combination of cefoperazone/sulbactam at a 1:1 ratio had better in vitro activity against most multiresistant organisms (including ESBL-producing *E. coli*, carbapenem-resistant *E. coli*, and carbapenem-resistant *A. baumannii*) compared to cefoperazone/sulbactam at a 2:1 ratio [7]. The study by Lai CC et al. showed that cefoperazone/sulbactam 1:1 had the highest in vitro susceptibility rates against CRAB, followed by cefoperazone/sulbactam 2:1 and cefoperazone (with susceptibility rates of 80.0%, 40.0%, and 0%, respectively). The susceptibility rates of CRAB for cefoperazone/sulbactam 1:2, 1:1, and 2:1 were 92.6%, 76.2%, and 41.0%, respectively. The author concluded that the in vitro susceptibility rates against CRAB can be enhanced by adding sulbactam to the cefoperazone treatment [57].

Cefoperazone/sulbactam-based therapeutic regimens have clinical effectiveness in patients with multiresistant *Acinetobacter baumannii* infections. Wang Q. et al. conducted a retrospective analysis among patients with MDRAB pneumonia and identified microbiological changes before and after therapy to determine the microbiological efficacy. The findings demonstrated that independent of tigecycline administration, the cefoperazone/sulbactam 3 g/q8h (the sulbactam dose was calculated as 3 g) regimen decreased the microbiological efficacy in MDRAB. The microbiological efficacy showed a noteworthy enhancement following an increase in the sulbactam dosage to 4 g or beyond. When treating MDRAB pneumonia, the cefoperazone/sulbactam-based anti-infective regimen showed some success. However, with time, the microbiological efficacy of the cefoperazone/sulbactam 3 g q8h regimen declined. The effectiveness may be enhanced by increasing the sulbactam dosage to 4 g or higher [58]. The study did not demonstrate whether the decreasing mortality and increasing clinical improvement were enhanced by raising the sulbactam dosage to 4 g or higher. As we know, a higher dosage of sulbactam does not always result in more antibacterial activity. From the literature, we still could not determine the most effective combination ratios of cefoperazone to sulbactam. Nevertheless, what ratio of cefoperazone to sulbactam inhibits multiresistant *Acinetobacter baumannii* the most remains unknown.

The meta-analysis by Ni W. et al. (2016) showed that treating MDRAB infections with tigecycline-based treatments may not be the optimal course of action. In their study, there was no significant difference in all-cause mortality and the clinical response of individuals treated with tigecycline compared to those receiving other antibiotics [34]. According to an in vitro activity study by Liu B et al., tigecycline with cefoperazone/sulbactam was more efficient against XDRAB than tigecycline plus sulbactam alone. Tigecycline and cefoperazone/sulbactam had synergistic effects against MDRAB in vitro, indicating that this combination might be a better therapeutic choice [59]. Forty-two patients with ventilator-associated pneumonia caused by XDRAB infection were included in a study by Qin Y et al. The patients were randomly assigned to one of two groups: the tigecycline or tigecycline plus cefoperazone/sulbactam groups. The tigecycline plus cefoperazone/sulbactam group showed a better overall combined effectiveness (85.7% effective) than the tigecycline group (47.6% effective). The author concluded that the antibacterial activity of tigecycline against XDRAB ventilator-associated pneumonia can be enhanced by adding cefoperazone/sulbactam [16]. In a study by Gu S et al., 15 (25.4%) of the 59 patients with a CRAB bloodstream infection who received cefoperazone/sulbactam-based therapy—which included both cefoperazone/sulbactam monotherapy and tigecycline in combination with cefoperazone/sulbactam therapy—died within 30 days after starting treatment. In addition, 22 (51.2%) of the 43 patients with CRAB bloodstream infections who received therapy that did not include cefoperazone/sulbactam died within 30 days, and the therapy options included carbapenem monotherapy, tigecycline monotherapy, and other regimens. The 30-day mortality rates for patients with a CRAB bloodstream infection who received decisive therapy with cefoperazone/sulbactam-based therapeutic regimens were considerably lower than those of patients who received non-cefoperazone/sulbactam-based therapeutic regimens [22]. In a study by Kanchanasuwan S et al., in patients with CRAB ventilator-associated pneumonia, the cefoperazone/sulbactam-based therapeutic regimens led to considerably lower thirty-day and in-hospital mortality rates than the non-cefoperazone/sulbactam-based therapeutic regimens, with respective values of 35% and 39% and 61% and 68% for the cefoperazone/sulbactam-based therapeutic regimens and non-cefoperazone/sulbactam-based therapeutic regimens. When comparing cefoperazone/sulbactam-based therapeutic regimens and non-cefoperazone/sulbactam-based therapeutic regimens, the survival rate for the former group was considerably higher [21]. In a study by Niu T et al., in patients with CRAB bloodstream infections, cefoperazone/sulbactam-based therapy was administered to 75 patients, while tigecycline-based therapy was given to 135 patients. Patients in the cefoperazone/sulbactam-based therapy group had a considerably lower 28-day mortality rate [29.3% vs. 51.9%; *p* = 0.001] than those in the tigecycline group. The cefoperazone/sulbactam-based therapy improved patient prognosis, according to the Cox multivariate regression analysis (*p* = 0.028) [18]. According to Xia et al., among patients with hospital-acquired pneumonia caused by CRAB, the 30-day survival rate of those treated with cefoperazone/sulbactam or a cefoperazone/sulbactam combination regimen was significantly higher than that of the patients who had not received cefoperazone/sulbactam (96.4% vs. 73.3%, respectively; *p* < 0.05). When cefoperazone/sulbactam was combined with levofloxacin, meropenem, and minocycline, it showed an additive and synergistic bacteriostatic effect on CRAB in vitro [9]. There is no doubt that the cefoperazone/sulbactam-based therapeutic regimens from several in vitro activity studies show that the in vitro activity against multiresistant *Acinetobacter baumannii* can be enhanced by adding sulbactam to cefoperazone. The effect is even better than that of ampicillin plus sulbactam [55]. However, there are very few clinical trials in the literature, and there is a lack of high-quality randomized controlled trials. Our meta-analysis showed that the cefoperazone/sulbactam-based therapeutic regimens are superior to the non-cefoperazone/sulbactam-based therapeutic regimens in terms of 30-day mortality and clinical improvement in patients with multiresistant *Acinetobacter baumannii* infections. This meta-analysis is a pioneering preliminary report. We hope this study can inspire others and allow medical experts to conduct further randomized controlled trials to confirm the role of cefoperazone/sulbactam-based therapeutic regimens in the treatment of multiresistant *Acinetobacter baumannii* infections.

### Limitations

There are many limitations to our meta-analysis, the majority of which are related to those present in the literature. Remarkably, only a few studies—none of which were randomized controlled trials—met our inclusion criteria. As a result, the quality of data that was taken for this research was subpar. Another drawback was the very small sample size of the studies that we included in our meta-analysis, which naturally lowers the statistical power. A lack of uniformity in the pharmaceuticals used in conjunction with sulbactam, the comparator medications, and the initial characteristics of patients are among the other drawbacks. Understanding the differences between the drugs taken with sulbactam and the comparison drugs may be difficult because some of these medicines have different pharmacokinetic and pharmacodynamic properties. More carefully monitored research is required.

## 5. Conclusions

To summarize, our meta-analysis results indicate that the treatments based on cefoperazone/sulbactam regimens were superior to the treatments based on non-cefoperazone/sulbactam regimens in patients with multiresistant *Acinetobacter baumannii* infections. The risk of mortality was reduced by 38% among patients who received the cefoperazone/sulbactam-based therapeutic regimens. We recommended cefoperazone/sulbactam-based therapeutic regimens in conjunction with one additional antimicrobial agent for the treatment of multiresistant *Acinetobacter baumannii* infections.

## 6. Future Directions

Our results emphasize the need for randomized, well-monitored clinical studies to ascertain the significance of findings.

## Figures and Tables

**Table 1:** Characteristics of the included studies.

Author/Year	Region/ Study Type	Bacteria	Infection Type	No of Patients (CPZ/SUL)	No of Patients (Non-CPZ/SUL)	Drug Dosage of CPZ/SUL	Drugs of Non-CPZ/SUL Groups	Combination Drugs of CPZ/SUL Groups
Xia J/2014 [9]	China/ RET	CRAB	HAP	41	30	No data	No data	Minocycline Meropenem, Levofloxacin
Li Y/2014 [14]	China RET	XDRAB	Mixed	15	21	CPZ/SUL (2:1) 3.0 gm q12h	Carbapemen	Carbapenem, Minocycline
Li P/2016 [15]	China/ RET	MDRAB XDRAB	HAP	35	71	No data	Carbapenem, Tigecycline	No antibiotic
Qin Y/2017 [16]	China/ RET	XDRAB	VAP	21	21	No data	Tigecycline	Tigecycline
Zhou H/2019 [17]	China/ RET	MDRAB	BSI	112	162	No data	Tigecycline	Tigecycline
Niu T/2019 [18]	China/ RET	CRAB	BSI	75	135	CPZ/SUL (2:1) 3.0 gm q8h–6.0 gm q6h	Tigecycline	Carbapenem
Gulin TA/2020 [19]	Turkey/ RET	MDRAB XDRAB	Mixed	11	96	No data	Colistin, Carbapenem, Tigecycline	Colistin
Yu K/2021 [20]	China/ RET	MDRAB	BSI	42	36	No data	Polymyxin B, Carbapenem, Tigecycline	Tigecycline
Kanchanasuwan S/2021 [21]	Thailand/ RET	CRAB	VAP	52	28	CPZ/SUL (1:1) 2.0 gm q8h–q4h	Imipenem, Tigecycline, Other antibiotics	Imipenem, Tigecycline, Other antibiotics
GU S/2023 [22]	China/ RET	CRAB	BSI	59	43	CPZ/SUL (2:1) 3.00 gm q8h–q6h	Carbapenem, Tigecycline, Other antibiotics	Tigecycline

Footnotes: RET: retrospective study; CRAB: carbapenem-resistant *Acinetobacter baumannii*; XDRAB: extensively drug-resistant *Acinetobacter baumannii*; MDRAB: multidrug-resistant *Acinetobacter baumannii*; HAP: hospital-acquired pneumonia; VAP: ventilator-associated pneumonia. BSI: bloodstream infection; CPZ: cefoperazone; SUL: sulbactam.

**Table 2:** Risk bias of ten studies.

Author/Year	Confounding	Selection	Interventions Classification	Interventions Deviations	Missing Data	Measurement of Outcomes	Selective Results
Xia J/2014 [9]	moderate risk	high risk	moderate risk	moderate risk	serious risk	serious risk	high risk
Li Y/2014 [14]	low risk	moderate risk	low risk	low risk	low risk	moderate risk	moderate risk
Li P/2016 [15]	moderate risk	moderate risk	high risk	high risk	serious risk	serious risk	serious risk
Qin Y/2017 [16]	high risk	high risk	high risk	high risk	serious risk	serious risk	serious risk
Zhou H/2019 [17]	low risk	low risk	low risk	moderate risk	serious risk	serious risk	high risk
Niu T/2019 [18]	low risk	low risk	low risk	moderate risk	low risk	low risk	low risk
Gulin TA/2020 [19]	high risk	high risk	moderate risk	high risk	serious risk	serious risk	serious risk
Yu K/2021 [20]	low risk	low risk	moderate risk	moderate risk	serious risk	serious risk	high risk
Kanchanasuwan S/2021 [21]	low risk	low risk	moderate risk	moderate risk	low risk	low risk	moderate risk
GU S/2023 [22]	low risk	low risk	moderate risk	moderate risk	low risk	low risk	low risk

## Data Availability

The datasets generated during and/or analyzed during the current study are not publicly available but are available from the corresponding author upon reasonable request.
